# Doxycycline attenuates breast cancer related inflammation by decreasing plasma lysophosphatidate concentrations and inhibiting NF-κB activation

**DOI:** 10.1186/s12943-017-0607-x

**Published:** 2017-02-08

**Authors:** Xiaoyun Tang, Xianyan Wang, Yuan Y. Zhao, Jonathan M. Curtis, David N. Brindley

**Affiliations:** 1 Department of Biochemistry, Signal Transduction Research Group, University of Alberta, Edmonton, AB T6G 2S2 Canada; 2 Department of Agricultural, Food and Nutritional Science, University of Alberta, 410 Agriculture/Forestry Centre, 3-60D South Academic Building, Edmonton, AB T6G 2P5 Canada; 3 Department of Biochemistry, 357 Heritage Medical Research Centre, University of Alberta, Edmonton, AB T6G 2S2 Canada

**Keywords:** Autotaxin, Inflammatory cyotokines, Tetracyclines, Peripheral blood mononuclear cells, Macrophage infiltration

## Abstract

**Background:**

We previously discovered that tetracyclines increase the expression of lipid phosphate phosphatases at the surface of cells. These enzymes degrade circulating lysophosphatidate and therefore doxycycline increases the turnover of plasma lysophosphatidate and decreases its concentration. Extracellular lysophosphatidate signals through six G protein-coupled receptors and it is a potent promoter of tumor growth, metastasis and chemo-resistance. These effects depend partly on the stimulation of inflammation that lysophosphatidate produces.

**Methods:**

In this work, we used a syngeneic orthotopic mouse model of breast cancer to determine the impact of doxycycline on circulating lysophosphatidate concentrations and tumor growth. Cytokine/chemokine concentrations in tumor tissue and plasma were measured by multiplexing laser bead technology. Leukocyte infiltration in tumors was analyzed by immunohistochemistry. The expression of IL-6 in breast cancer cell lines was determined by RT-PCR. Cell growth was measured in Matrigel™ 3D culture. The effects of doxycycline on NF-κB-dependent signaling were analyzed by Western blotting.

**Results:**

Doxycycline decreased plasma lysophosphatidate concentrations, delayed tumor growth and decreased the concentrations of several cytokines/chemokines (IL-1β, IL-6, IL-9, CCL2, CCL11, CXCL1, CXCL2, CXCL9, G-CSF, LIF, VEGF) in the tumor. These results were compatible with the effects of doxycycline in decreasing the numbers of F4/80^+^ macrophages and CD31^+^ blood vessel endothelial cells in the tumor. Doxycycline also decreased the lysophosphatidate-induced growth of breast cancer cells in three-dimensional culture. Lysophosphatidate-induced Ki-67 expression was inhibited by doxycycline. NF-κB activity in HEK293 cells transiently expressing a NF-κB-luciferase reporter vectors was also inhibited by doxycycline. Treatment of breast cancer cells with doxycycline also decreased the translocation of NF-κB to the nucleus and the mRNA levels for IL-6 in the presence or absence of lysophosphatidate.

**Conclusion:**

These results contribute a new dimension for understanding the anti-inflammatory effects of tetracyclines, which make them potential candidates for adjuvant therapy of cancers and other inflammatory diseases.

**Electronic supplementary material:**

The online version of this article (doi:10.1186/s12943-017-0607-x) contains supplementary material, which is available to authorized users.

## Background

Chronic inflammation is one of the intrinsic features of the tumor microenvironment, which makes it an important hallmark of cancer development and progression [1–3]. Autotaxin (ATX) is a secreted enzyme, which is a key regulator of inflammation through its production of lysophosphatidate (LPA) from lysophosphatidylcholine [4, 5]. LPA signals through six G protein-coupled receptors to stimulate cell proliferation, survival, migration and angiogenesis, which promote tumor growth [4, 6]. Increased ATX and LPA levels are also important in the development of chronic inflammation in asthma, pulmonary fibrosis, rheumatoid arthritis, atherosclerosis, hepatitis, multiple sclerosis, Crohn’s disease and ulcerative colitis [7–9].

Increased LPA signaling is closely associated with tumor growth and cancer-related inflammation [10–13]. This is because LPA induces the expression of inflammatory cytokines through activating nuclear factor-κB (NF-κB) [14, 15]. We reported that inflammatory cytokines from breast tumors stimulate ATX production by adjacent adipose tissue [11]. The consequently high LPA concentration enhances lymphocyte infiltration, which increases the inflammatory status in the tumor. This vicious cycle of LPA signaling increases the production of inflammatory mediators, which further increases tumor growth, metastasis and the development of chemo-resistance [10, 11, 16]. Cancer-related inflammation comprises a complicated crosstalk between cancer cells and leukocytes. The massive infiltration of tumor associated macrophages (TAM), especially M2 macrophages, represents a poor prognosis in many types of cancer [17–19]. In the tumor microenvironment, TAMs originate from the monocyte lineage through the action of cytokines, e.g., chemokine (C-C motif) ligand 2 (CCL2), secreted by stromal cells and cancer cells [20, 21]. TAMs stimulate cancer cell proliferation by secreting growth factors, e.g., epidermal growth factor (EGF) [22] and platelet derived growth factor (PDGF) [23]. TAMs also promote angiogenesis by producing vascular endothelial growth factor (VEGF) [24] and suppress antitumor immunity by secreting immune regulatory molecules such as IL-10 [25] and transform growth factor β (TGFβ) [26].

Treatment of mice that had breast cancer with a specific ATX inhibitor, ONO-8430506, had an anti-inflammatory effect. It decreased LPA level in plasma and tumors, thereby decreasing the concentrations of 20 inflammatory cytokines/chemokines in adipose tissue adjacent to the tumor [10, 27]. An alternative way to regulate LPA levels is through a family of enzymes named lipid phosphate phosphatases (LPPs), which consists of three isoforms, LPP1, LPP2 and LPP3 [28, 29]. LPPs dephosphorylate extra-cellular LPA to monoacylglycerol, which terminates LPA signaling. The expressions of LPP1 and LPP3 are decreased in many cancers, including breast cancer [28, 30]. We recently discovered that tetracyclines increase extracellular LPA degradation by enhancing the stabilities of LPP1, LPP2 and LPP3 in several breast cancer cell lines and in non-transformed cells [31]. The clearance of LPA from the circulation in rats was accelerated by doxycycline treatment and LPA concentrations in mouse plasma were decreased [31]. This tetracycline effect does not involve the inhibition of matrix metalloproteinase activity [31].

Tetracyclines also show anti-inflammatory effects, and their clinical use has been expanded from microbial infection to inflammatory diseases including acne [32], rosacea [33], perioral dermatitis [34] and gingivitis [35]. Effective use of tetracyclines has been reported in rheumatoid arthritis [36], osteoarthritic cartilage [37], allergen-induced inflammation and inflammatory skin disorders [38].

Therefore, the effects of tetracyclines on LPA degradation and inflammation suggest that they may have beneficial effects on cancer therapy. In the present study, we demonstrated that doxycycline decreased breast tumor growth in a syngeneic orthotopic mouse model. Doxycycline treatment decreased plasma LPA levels and the concentrations of several inflammatory mediators, the infiltration of F4/80^+^ macrophages and blood vessel formation in the tumor. Doxycycline also inhibited NF-κB activation in breast cancer cells by decreasing phosphorylation of the inhibitor of κB (IκB) and nuclear translocation of NF-κB. These results demonstrate that doxycycline has a novel action in decreasing LPA signaling, which contributes to its anti-inflammatory effects. These actions provide new mechanisms that support the use of tetracyclines as an adjuvant therapy for cancers and other inflammatory diseases.

## Results

### Doxycycline delayed tumor growth and decreased the numbers of tumor-associated macrophages and blood vessels in a syngeneic orthotopic mouse model of breast cancer

We recently discovered that doxycycline increased the dephosphorylation of extracellular LPA in MDA-MB-231, MCF-7 and 4T1 breast cancer cells by increasing the expression of the LPPs on the cell surface [31]. This explained why animals treated with doxycycline showed increased clearance of LPA from the circulation and decreased plasma LPA levels. These results indicate that doxycycline could have favorable effects on cancer treatment, since LPA signaling is up-regulated in many cancers.

**Fig. 1:**
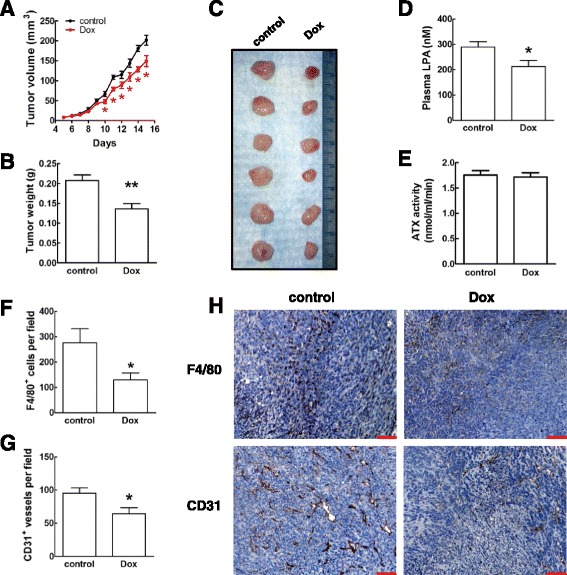
Doxycycline delayed breast tumor growth, decreased plasma LPA concentration, inhibited F4/80^+^ macrophage infiltration and blood vessel formation in the tumor. **a**. Tumor volume from day 5 to day 15 post inoculation of 4T1 cells. BALB/c mice were treated with doxycycline (Dox) at 50 mg/kg/day by i.p. injection. Control mice were given saline by i.p. injection. *n* = 6 for each group, * *p* < 0.05 relative to control. **b**. The difference in tumor weight. *n* = 6 for each group, ** *p* < 0.01 relative to control. **c**. Image of the tumors from control and Dox treated mice. **d**. Plasma LPA concentration of the mice with tumor. *n* = 6 for each group, * *p* < 0.05 relative to control. **e**. Plasma autotoxin (ATX) activity of control and Dox treated mice. **f**. F4/80^+^ macrophage numbers per field detected by IHC in tumors from control and Dox treated mice. * *p* < 0.05 relative to control. **g**. CD31^+^ blood vessel numbers per field detected by IHC in tumors from control and Dox treated mice. * *p* < 0.05 relative to control. These were quantified by examining 5 different fields from each tumor and by using 6 mice per group. **h**. Representative images of IHC staining for F4/80^+^ macrophages and CD31^+^ blood vessels in tumors from control and Dox treated mice. Scale bar = 100 μm. Results are means ± SEM. Results were analyzed by a Student’s *t*-test

We, therefore, used a syngeneic mouse model of breast cancer to study the effects of doxycycline on tumor growth. Doxycycline at 50 mg/kg/day was tolerated fairly well and it resulted in a loss of body weight of only ~10% after 15 days. Doxycycline treatment significantly decreased tumor volume by ~25% and tumor weight by ~35%. (Fig. 1a, b). There was also a significant decrease in tumor weight in the doxycycline-treated group of ~20% when it was expressed relative to body weight (Additional file 1: Figure S1A). The inhibitory effect of doxycycline on tumor growth was maintained for 24 days after inoculation (Additional file 1: Figure S1B). The apparent decrease in the number of metastatic nodules on lung surface 24 days after inoculation in doxycycline-treated mice did not reach the level of statistical significance (Additional file 1: Figure S1C). As predicted, doxycycline treatment decreased plasma LPA concentrations by ~26% (Fig. 1d), which can be explained by the doxycycline-induced increase of LPP activity on the cells surface [31]. ATX activity in plasma was not affected significantly by doxycycline (Fig. 1e).

Immunohistochemistry staining of the breast tumors demonstrated that doxycycline treatment significantly decreased the numbers of F4/80^+^ macrophages by ~50% and CD31^+^ blood vessels in the tumor by ~30% (Fig. 1f, g and h). There was no significant change in infiltration of total CD45^+^ leukocytes, CD8^+^ cytotoxic T cells and Foxp3^+^ regulatory T cells (Additional file 1: Figure S1D).

### Doxycycline decreased inflammatory cytokine levels in plasma and tumor tissue

**Fig. 2:**
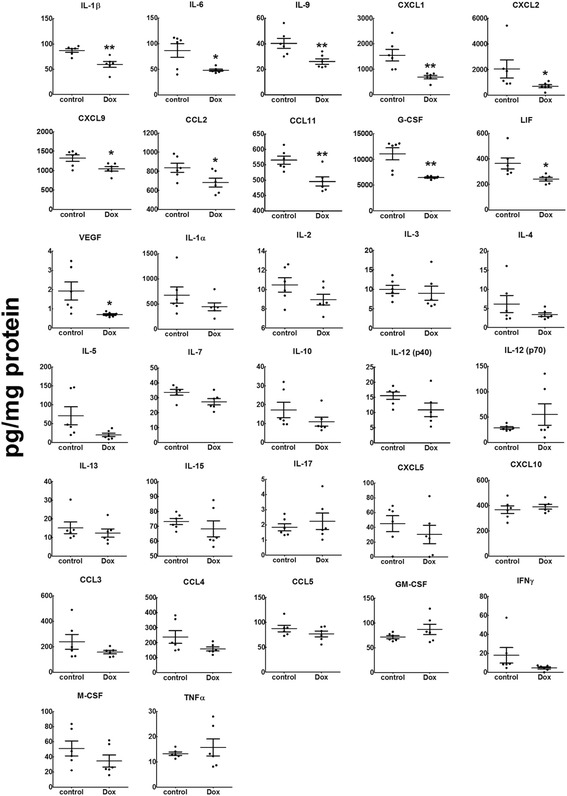
BALB/c mice treated with Doxycycline (Dox) at 50 mg/kg/day showed significantly decreased concentrations of IL-1β, IL-6, IL-9, CXCL1, CXCL2, CXCL9, CCL2, CCL11, G-CSF, LIF and VEGF in the tumor. Concentrations of IL-1α, IL-2, IL-3, IL-4, IL-5, IL-7, IL-10, IL-12 (p40), IL-12 (p70), IL-13, IL-15, IL-17, CXCL5, CXCL10, CCL3, CCL4, CCL5, GM-CSF, IFNγ, M-CSF and TNFα were not affected by Dox. *n* = 6 for each group. Results are means ± SEM, * *p* < 0.05, ** *p* < 0.01 relative to control. Results were analyzed by a Student’s *t*-test

Since LPA is an important mediator of inflammation, we next determined the levels of cytokines in the breast tumor. Doxycycline treatment significantly decreased the concentrations of IL-1β, IL-6, IL-9, CXCL1, CXCL2, CXCL9, CCL2, CCL11, G-CSF, LIF, and VEGF in the tumor (Fig. 2). In addition, we measured the concentrations of G-CSF, IL-1β, IL-6, CCL4 and TNFα in the plasma of the mice with breast cancer. Only G-CSF was decreased significantly by doxycycline treatment (Additional file 2: Figure S2).

### Doxycycline decreased IL-6, CCL2 and CXCL2 expression in 4T1 cells

**Fig. 3:**
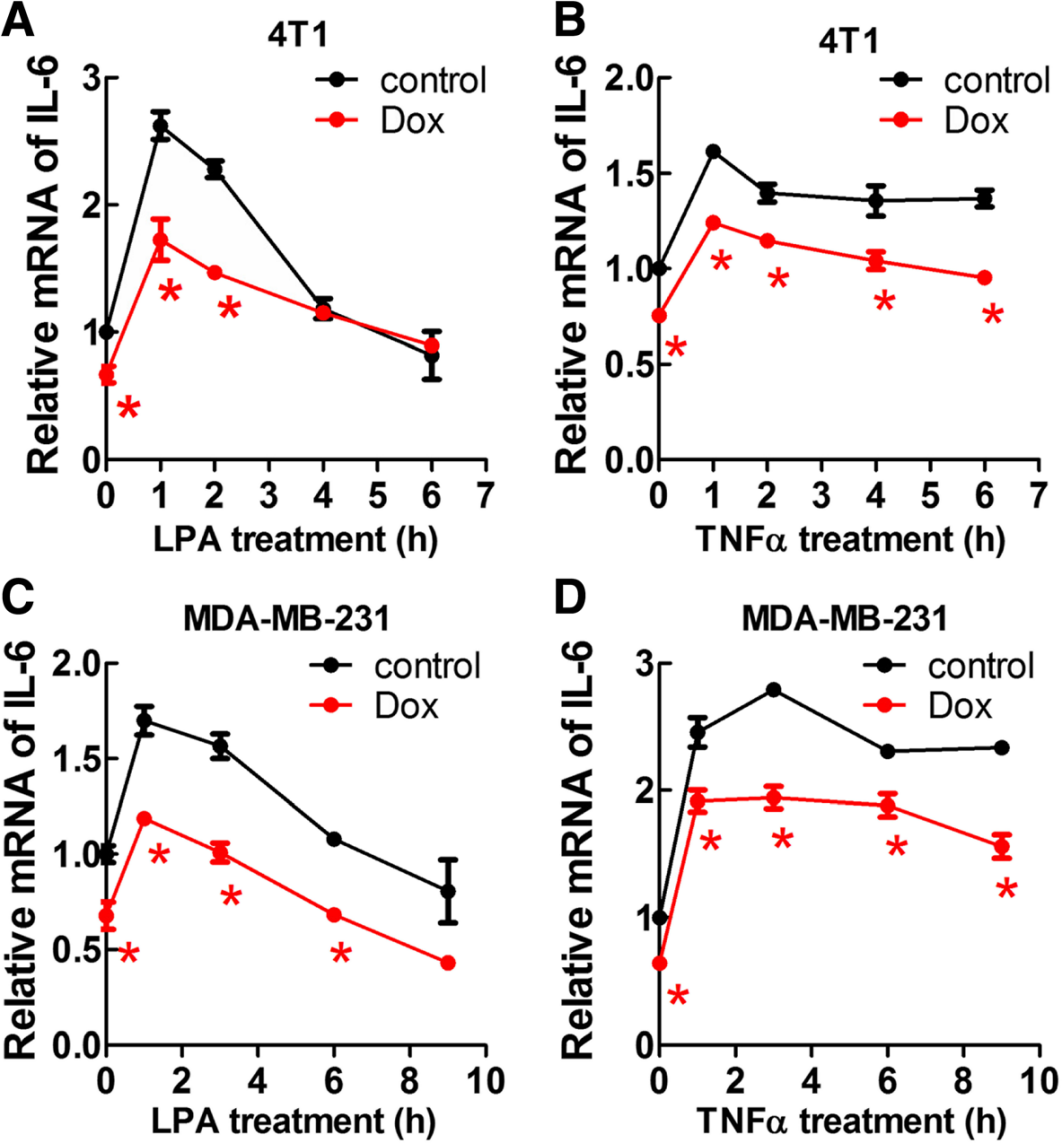
Doxycycline (Dox) significantly decreased LPA and TNFα induced IL-6 mRNA expression in breast cancer cells. Cells were serum starved for 14 h in DMEM/0.1% BSA and then followed with LPA (1 μM) or TNFα (20 ng/ml) stimulation. For Dox-treated cells, 5 μg/ml of Dox was included during serum starvation and stimulation. **a**. Time course of LPA induced IL-6 expression in 4T1 cells and the inhibition by Dox. **b**. Time course of TNFα induced IL-6 expression in 4T1 cells and the inhibition by Dox. **c**. Time course of LPA induced IL-6 expression in MDA-MB-231 cells and the inhibition by Dox. **d**. Time course of TNFα induced IL-6 expression in MDA-MB-231 cells and the inhibition by Dox. * *p* < 0.05 relative to control. Results are means ± SEM from three independent experiments. Results were analyzed by ANOVA with an SNK posthoc test

**Fig. 4:**
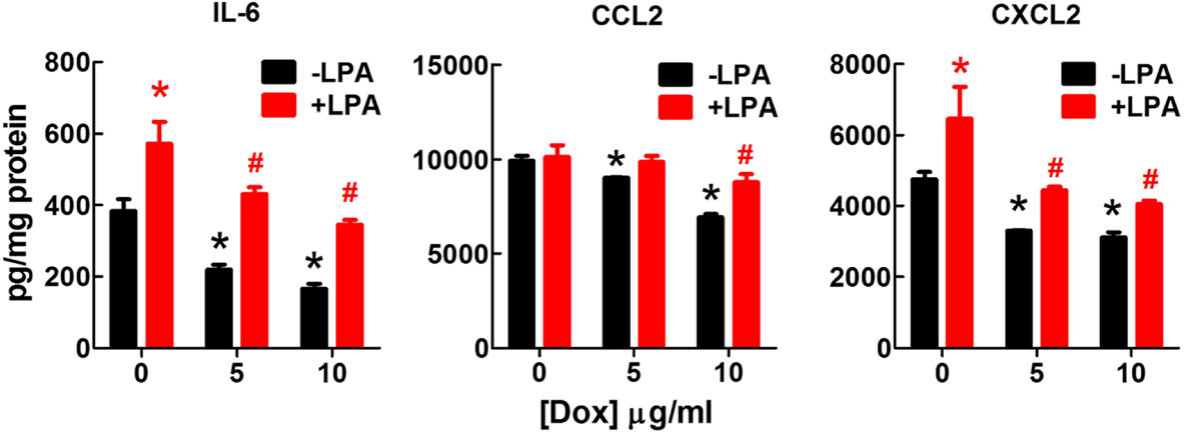
Doxycycline (Dox) significantly decreased the secretion of IL-6, CCL2 and CXCL2 by 4T1 cells. Cells were cultured with DMEM/10% FBS. The medium was changed with DMEM/0.1% BSA with or without LPA (5 μM) and Dox (5 μg/ml or 10 μg/ml). Conditioned medium was collected after incubation for another 24 h. Measurements were normalized to the cell protein. * *p* < 0.05 relative to cells without Dox and LPA treatment, # *p* < 0.05 relative to cells treated with LPA but not Dox. Results are means ± SEM from three independent experiments. Results were analyzed by ANOVA with an SNK posthoc test

Tumors are composed of cancer and stromal cells, all of which express cytokines. Our previous work showed that LPA stimulated inflammatory cytokine secretion by mouse 4T1 breast cancer cells [11]. Doxycycline decreased plasma LPA [31], which could cause lower cytokine production by cancer cells. In present study, LPA induced a rapid increase of IL-6 mRNA expression, which reached a peak at 1 h after stimulation of 4T1 cells. Doxycycline treatment decreased IL-6 expression, even when LPA was absent (Fig. 3a). This indicated that the inhibition of IL-6 expression by doxycycline was not entirely through suppressing LPA signaling. Therefore, we used TNFα as an agonist and doxycycline also inhibited TNFα-induced IL-6 expression (Fig. 3b). Similar results were observed in human MDA-MB-231 cells, in which doxycycline decreased the expressions of IL-6 in the presence or absence of LPA or TNFα (Fig. 3c and d). We also showed that doxycycline decreased the secretions of IL-6, CCL2 and CXCL2 by 4T1 cells and this did not depend on the presence of LPA (Fig. 4).

### Doxycycline decreased NF-κB translocation and IκB phosphorylation in breast cancer cells

**Fig. 5:**
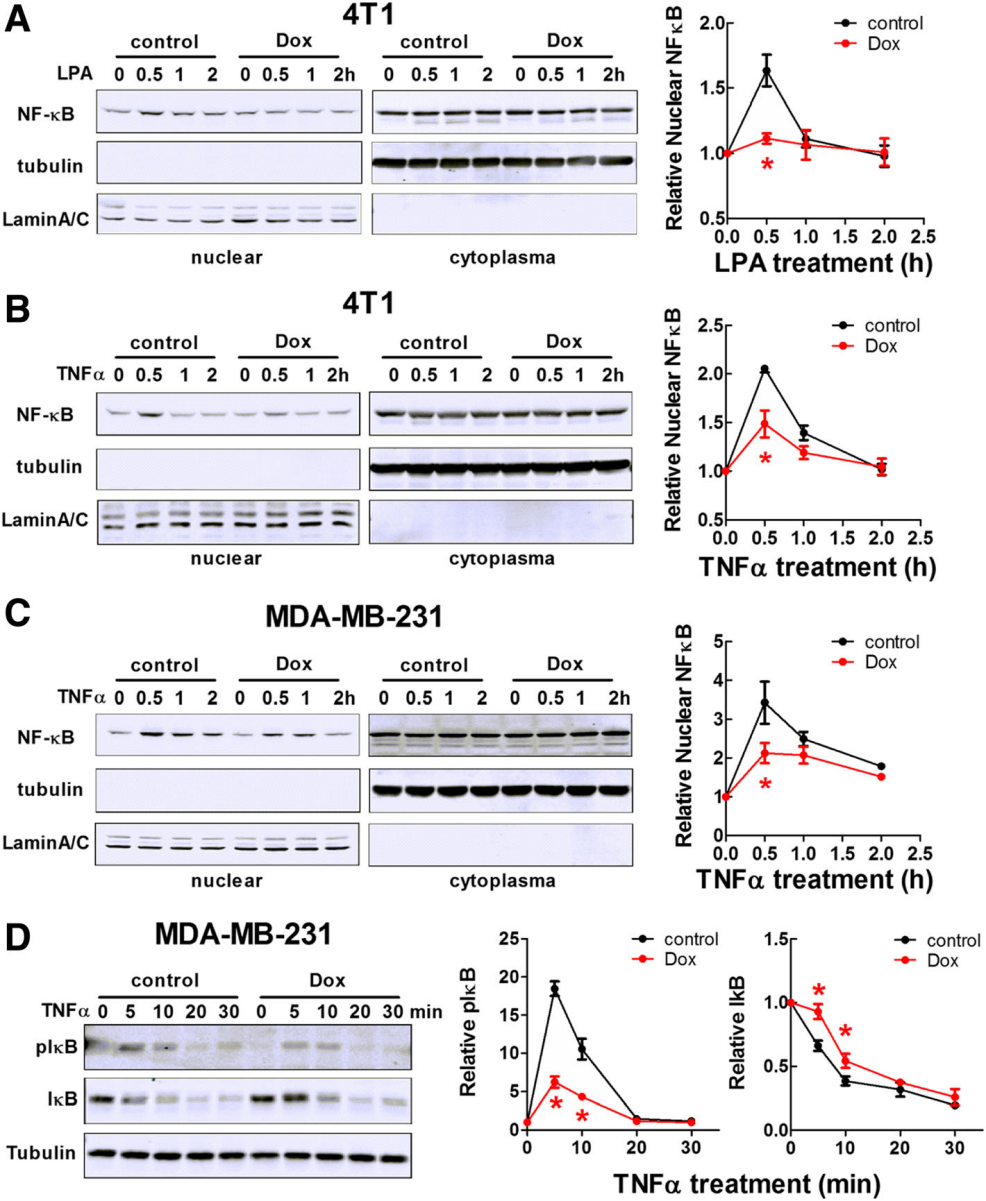
Doxycycline (Dox) inhibited LPA and TNFα-induced translocation of NF-κB p65 to the nucleus in breast cancer cells. Cells were serum starved for 14 h in DMEM/0.1% BSA and then followed stimulation with 5 μM LPA or 20 ng/ml TNFα. For Dox-treated cells, 5 μg/ml of Dox was included during serum starvation and stimulation. **a**. Time course of NF-κB translocation to nucleus induced by LPA in 4T1 cells and the effect of Dox. **b**. Time course of NF-κB translocation to nucleus induced by TNFα in 4T1 cells and the effect of Dox. **c**. Time course of NF-κB translocation to nucleus induced by TNFα in MDA-MB-231 cells and the effect of Dox. **d**. The effect of Dox on the time course of phospho-IκBα and total IκBα by TNFα in MDA-MB-231 cells. * *p* < 0.05 relative to control. Results are means ± SEM from three independent experiments. Results were analyzed by ANOVA with an SNK posthoc test

Inflammatory signals from both LPA and TNFα receptors can converge on NF-κB, which increases the expression of inflammatory cytokines, e.g., IL-6. We predicted that doxycycline decreases inflammatory cytokine expression by inhibiting NF-κB-mediated transcription. As expected, LPA and TNFα increased the translocation of NF-κB to the nucleus in 4T1 cells. These effects were inhibited by doxycycline (Fig. 5a and b). Doxycycline also inhibited TNFα-induced translocation of NF-κB to the nucleus in MDA-MB-231 cells (Fig. 5c). In agreement with this, IκB phosphorylation and the degradation of total IκB induced by TNFα were decreased by doxycycline (Fig. 5d). Doxycycline decreased both the basal and TNFα-induced ratio of luminescence in HEK293 cells transiently transfected with NF-κB luciferase reporter and Renilla luciferase vectors (Additional file 3: Figure S3). Therefore, the anti-inflammation effect of doxycycline involves decreases in NF-κB-induced transcription.

### Doxycycline inhibited migration of mouse peripheral blood mononuclear cells (PBMCs) and IκB phosphorylation in RAW264.7 cells

**Fig. 6:**
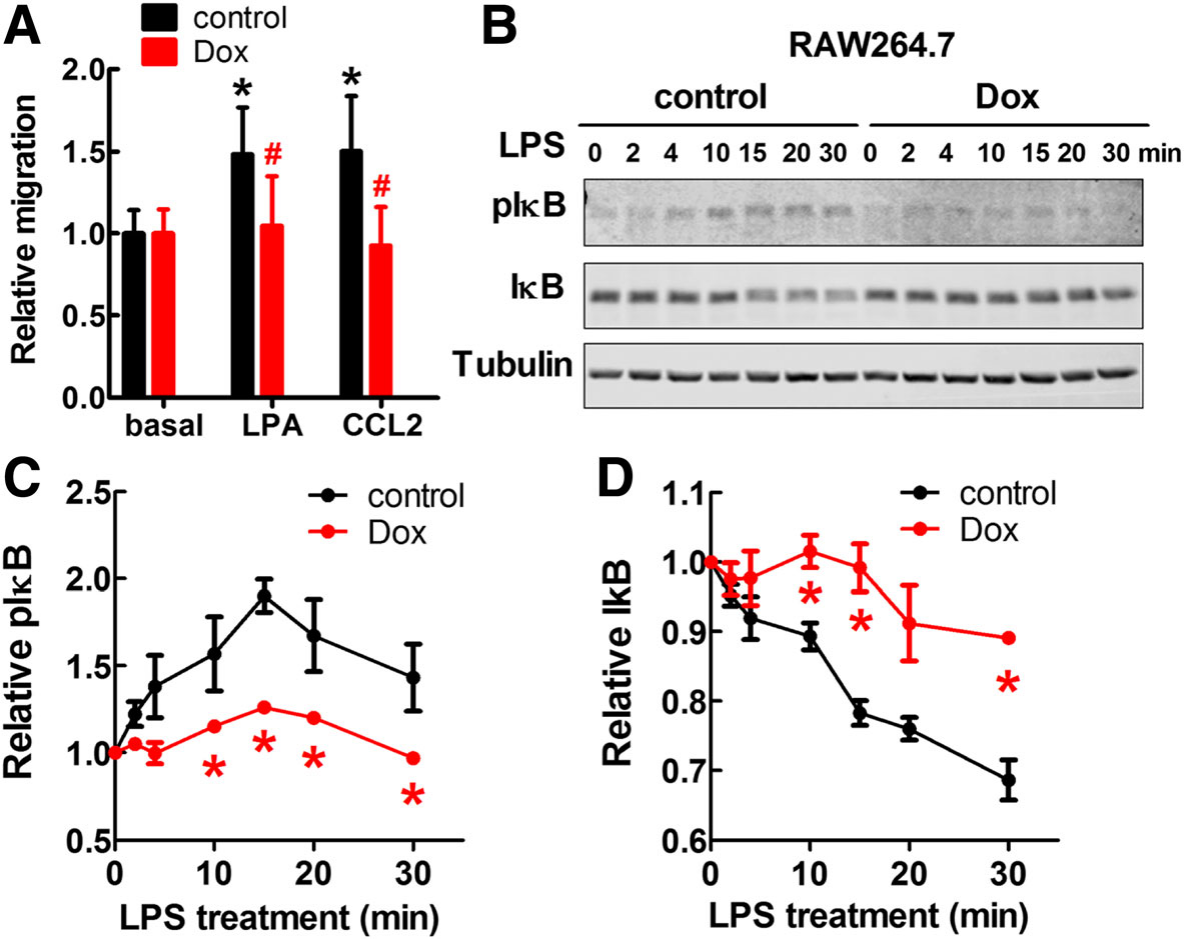
Effects of Doxycycline on PBMCs and RAW264.7 cells. **a**. Dox at 5 μg/ml suppressed the migration of mouse PBMCs induced by 1 μM of LPA and 100 ng/ml of CCL2. * *p* < 0.05 relative to basal level, # *p* < 0.05 relative to control. **b**. The effect of Dox on time course of phosphor-IκBα and total IκBα by 50 ng/ml of LPS in RAW264.7 cells. **c**. Quantification of phosphor-IκBα. **d**. Quantification of total IκBα. * *p* < 0.05 relative to control. Results are means ± SEM from three independent experiments. Results were analyzed by ANOVA with an SNK posthoc test

The inhibition of macrophage infiltration in tumors observed in Fig. 1d and e could have been caused by the doxycycline-induced decrease in the concentrations of chemo-attractants in the tumor (Fig. 2). It is also possible that doxycycline could directly inhibit the activation of macrophages [39]. We showed that doxycycline significantly decreased the migration of mouse PBMCs that was induced by LPA or CCL2 (Fig. 6a). Induction of IκB phosphorylation and degradation by lipopolysaccharide (LPS) in RAW264.7 macrophage cells were suppressed by doxycycline (Fig. 6b, c, d).

### Doxycycline did not affect LPA signaling downstream of LPA receptor activation

**Fig. 7:**
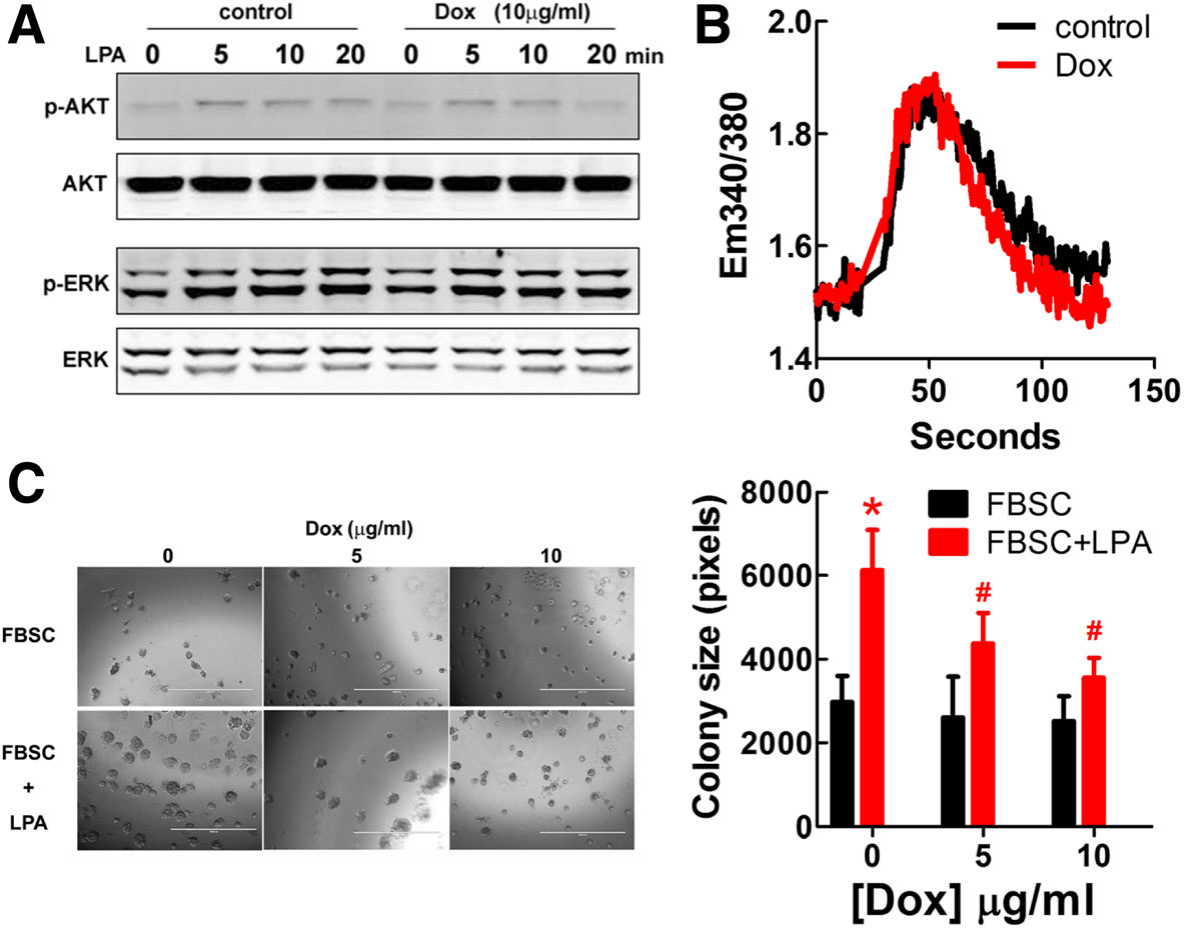
Doxycycline (Dox) did not affect signal transduction by transient LPA stimulation, but attenuated the long term effect of LPA on cell growth. **a**. 4T1 cells were serum starved with DMEM/0.1% BSA and stimulated with 1 μM LPA for 5, 10 and 20 min. Dox at 10 μg/ml was included in serum starvation and stimulation. LPA-induced stimulations of Akt and ERK phosphorylations were not affected by Dox. **b**. MDA-MB-231 cells were serum starved with DMEM/0.1% BSA and stimulated with 10 μM LPA. Dox at 10 μg/ml was included in serum starvation. LPA-induced Ca^2+^-mobilization was not affected by Dox. **c**. A three-dimensional  culture system was established by layering 400 μl of 4T1 cell suspension (6000 cells in DMEM with 10% FBSC and 2% Matrigel™) over 150 μl Matrigel™ in an 8-well chamber with daily replacement with fresh medium containing LPA or drugs. Dox decreased the stimulation of 4T1 cell colony formation by 5 μM LPA after incubation for 9 days. Scale bar = 1000 μm. *p < 0.05 relative to cells without Dox and LPA treatment, # *p* < 0.05 relative to cells treated with LPA but not Dox. Results are means ± SEM from three independent experiments. Results were analyzed by ANOVA with an SNK posthoc test

LPPs control signaling by two distinct mechanisms: 1) by decreasing extracellular LPA concentrations and 2) by degrading a lipid phosphate formed downstream of the activation of G protein coupled receptors, including LPA receptors [40]. To determine how doxycycline inhibits LPA signaling, we used LPA to induce the phosphorylations of Akt and ERK in 4T1 cells. Doxycycline at 10 μg/ml did not affect the phosphorylations of Akt or ERK (Fig. 7a). Similarly, Ca^2+^-transients induced by 10 μM of LPA were not changed by doxycycline in MDA-MB-231 cells (Fig. 7b).

Doxycycline does enhance LPA degradation outside of cells [31]. However, this is a relatively slow process, which would not be rapid enough to substantially decrease the availability of the 1 to 10 μM LPA used in the transient stimulations in Fig. 7a, b. By contrast, doxycycline did inhibit the effect of LPA in stimulating the proliferation of 4T1 cells in 3-D culture over 9 days where there was sufficient time each day to degrade extracellular LPA. The dependency of the doxycycline effect on LPA was established since cell growth induced by charcoal treated FBS (FBSC), in which LPA was severely depleted, was not affected by doxycycline (Fig. 7c). In monolayer culture, doxycycline also inhibited Ki-67 expression stimulated by LPA in MDA-MB-231 cells (Additional file 4: Figure S4). These results demonstrate that it is the doxycycline-induced expression of the LPPs on the cell surface [31], which decreases external LPA availability that is responsible for decreasing the LPA effects on cell growth.

## Discussion

Our previous study demonstrated a novel action of tetracyclines in increasing the stability of the LPPs in cancer and non-transformed cells [31]. This increased the expressions of LPP1, LPP2 and LPP3 at the surface of cells, which increased the degradation of extracellular LPA and lowered circulating LPA concentrations in mice. Up-regulations of ATX, LPA receptors and LPA levels coupled with decreased expression of LPP1 and LPP3 are closely associated with the growth and metastasis of many cancers [5, 28, 40]. Therefore, we determined if this novel effect of tetracyclines on LPP expression could decrease breast tumor growth. We showed that doxycycline decreased plasma LPA levels and delayed tumor growth in a syngeneic mouse model of breast cancer. LPA is also one of the critical triggers of tumor-induced inflammation by inducing the production of inflammatory cytokines in breast cancer cells [11]. In agreement with this, the doxycycline-induced decrease in plasma LPA was accompanied by a decrease in the concentrations of several cyotkines/chemokines (IL-1β, IL-6, IL-9, CCL2, CCL11, CXCL1, CXCL2, CXCL9, G-CSF, LIF, VEGF) in the tumor.

LPPs have two mechanisms for attenuating LPA signaling. First, LPPs on the plasma membrane degrade extracellular LPA, which decreases the amount of external LPA that can signal through its receptors [31]. Doxycycline specifically increases this ecto-activity of the LPPs, although it did not modify the rapid effects of LPA in activating Ca^2+^-transients, phosphorylations of ERK or Akt. This is explained since the ecto-LPP activity on plasma membranes would not have degraded sufficient LPA in our short-term experiments to attenuate rapid signaling. Doxycycline did, however, decrease the longer-term action of LPA in stimulating cell proliferation in 3D culture. The second mode of action of the LPPs is that increased expression of LPPs inside cells blocks cell signaling downstream of LPA and other G-protein coupled-receptors [28, 41]. This appears to involve the degradation of lipid phosphates formed downstream of receptor activation [42]. Consequently, targeted overexpression of LPP1 inside cells does attenuate LPA-induced activation of Ca^2+^-transients [30, 43]. In the case of human bronchial epithelial cells, this effect blocked the phosphorylation of IκB and translocation of NF-κB to the nucleus, which almost completely prevented IL-8 secretion [43]. However, this downstream effect on LPA receptor signaling was not involved in the doxycycline effect on LPP expression, which is increased LPP expression on the plasma membrane and thus decreased external LPA concentrations.

NF-κB activation is stimulated by different receptors, e.g., the toll like receptor family, the TNF receptor super family and G protein-coupled receptors, including LPA receptors [44]. The signals are transmitted through different pathways depending on the type of receptor activated, but they converge on IκB kinase (IKK) [45]. IKK phosphorylates IκB, an NF-κB inhibitor that prevents the translocation of NF-κB to the nuclear by binding to it in a dephosphorylated state. Upon phosphorylation, IκB is degraded through ubiquitination and this releases NF-κB from the sequestration. NF-κB then enters the nuclear and mediates the expression of genes for inflammatory cytokines [45]. Our study showed that doxycycline suppressed both LPA- and TNFα-induced nuclear translocation of NF-κB and blocked the LPA-induced secretion of IL-6, CCL2 and CXCL2 in cancer cells. TNFα-induced nuclear NF-κB transcriptional activity was also inhibited by doxycycline. Under basal condition without stimulation, doxycycline was able to decrease the transcriptional activity of nuclear NF-κB by ~50%, which explained the decreased IL-6 mRNA and secretion of IL-6, CCL2 and CXCL2 by doxycycline when LPA and TNFα were absent. However, the nuclear translocation of NF-κB and IκB phosphorylation were not affected significantly by doxycycline under this basal condition. Although NF-κB nuclear translocation is an important step in NFκB activation, modification of nuclear NF-κB by various events including phosphorylation, ubiquitination, nitrosylation, acetylation and interaction with different co-activators can affect its activity [46, 47]. Therefore, doxycycline inhibited the transcriptional activity of nuclear NF-κB, but did not affect nuclear translocation of NF-κB under basal condition. Upon stimulation, doxycycline was able to inhibit both the induced NF-κB translocation and transcriptional activity. These results establish that doxycycline inhibited NF-κB-mediated transcription independently of signaling by LPA. Therefore, the anti-inflammatory activity of doxycycline consists of at least two components: 1) A decrease in the availability of LPA for stimulating inflammation and 2) Inhibition of NF-κB activation that also decreases the production of inflammatory cytokines.

The chronic inflammatory milieu inside tumors polarizes TAMs to promote tumor growth [17–19]. For example, TAMs secrete growth factors including EGF [22], PDGF [23] and VEGF [24] to promote cancer cell proliferation and blood vessel formation. TAMs also inhibit the immune reaction of CD8^+^ T-cells against cancer cells by producing IL-10 and TGFβ [26]. In our work, doxycycline not only suppressed the cancer cell-derived chemo-attractants for monocytes, e.g., IL-6 and CCL2, but also directly impaired the migration activity of mouse PBMCs. These actions on both cancer cells and monocytes were compatible with the ~50% decrease of macrophage infiltration in the tumor. Furthermore, doxycycline blocked IκB phosphorylation and degradation in RAW264.7 macrophage cells in response to LPS, suggesting that the expressions of NF-κB target genes in TAMs were decreased. This could explain the different cytokine profile in the tumors after doxycycline treatment. Growth factors, e.g., G-CSF and VEGF, which are controlled by NF-κB [48, 49], were also decreased in the tumor by doxycycline. In many cancers, increased infiltration of tumors with TAMs is normally associated with a poor prognosis and the efficacy of targeting macrophages has been verified in preclinical studies for cancer therapy [50, 51]. The capability of doxycycline to selectively suppress TAM infiltration could maintain the anti-tumoral effects of CD8^+^ cytotoxic T-cells.

The effects of doxycycline that we reported in mice were obtained at a dose of 50 mg/kg/day. The equivalent dose for human is ~4 mg/kg/day calculated by equivalent surface area dosage conversion factor, which is 240 mg/day for 60 kg of body weight. The typical dose of doxycycline is 100–200 mg/day and the maximum dose is 300 mg/day for more serious infections, such as syphilis. Therefore, the dose of doxycycline used in this study is within the therapeutic range, which is used clinically.

## Conclusion

The present work established the importance of a novel dimension of tetracycline action. Doxycycline is a relatively inexpensive, commonly used and well-tolerated compound, which has multiple functions in addition to its anti-microbial activity. It is well known that tetracyclines inhibit matrix metalloproteinase activities [52]. These enzymes degrade extracellular matrix and this is involved in cancer cell invasion and migration. Several reports showed that tetracyclines have anti-neoplastic activity [51, 53, 54], but the mechanisms for this are poorly understood. This paper, together with our previous work [31], demonstrates a novel effect of tetracyclines in decreasing extracellular LPA concentrations by increasing the “ecto-activity” of the LPPs. This effect could be important for the treatment of cancers and other inflammatory conditions since LPA is an important regulator of inflammation through activation of NF-κB. In addition, doxycycline had a further action in decreasing NF-κB activation independently of the LPA signal. These combined actions of doxycycline make it a potential candidate for an adjuvant therapy for cancer and other inflammatory diseases.

## Methods

### Reagents and cell lines

Oleoyl-LPA (233019) was from Avanti Polar Lipids (Alabaster, AL). Doxycyclinehyclate (0219895525) was from MP Biomedicals (Solon, OH). Fatty acid-free albumin from bovine serum (A8806), mouse anti tubulin (T6074) antibody, probenecid (P8761), Calcein AM (C1359), OptiPrep™ density gradient medium (D1556), lipopolysaccharides (L3012) and protease inhibitor cocktail (P8340) were from Sigma (St. Louis, MO). Rabbit anti CD45 (ab10558), rabbit anti Foxp3 (ab54501), rabbit anti CD31 (ab28364), Alexa Fluor 488 conjugated anti rabbit IgG (ab150077) and HRP conjugated anti rat IgG (ab6734) antibodies were from Abcam (Toronto, ON, Canada); Rat anti F4/80 (14–4801), rat anti CD8α (14–0808) antibodies were from eBioscience (San Diego, CA); mouse anti phospho-Akt (4051), rabbit anti Akt (4691), mouse anti phospho-ERK (9106), rabbit anti ERK (9102), mouse anti IκB (4814), rabbit anti phosphor-IκB (Ser32) (2859), rabbit anti Ki-67 (D3B5) and rabbit anti NF-κBp65 (8242) antibodies were from Cell Signaling Technology (Danvers, MA); Rabbit anti Lamin A/C (sc-20681) was from Santa Cruz (Dallas, TX). Fura-2 AM (F-1201) and F127 (P-6867) were from Life Technologies (Grand Island, NY). HRP conjugated anti rabbit IgG antibody and DAB was from DAKO (Carpinteria, CA). Matrigel™ (354230/354234) was from Corning (Corning, NY). Recombinant human TNFα (Z100857), recombinant mouse TNFα (Z200217), reverse transcription master mix (G490) and EvaGreen qPCR master mix (MasterMix-ER) were from Applied Biological Materials Inc. (Richmond, BC, Canada). Human mammary carcinoma cell lines MDA-MB-231, mouse mammary carcinoma cell line 4T1, mouse macrophage cell line RAW264.7, and HEK (human embryonic kidney) 293 cells were from ATCC (Manassas, VA). Cells were cultured in Dulbecco’s Modified Eagle Medium (DMEM) with 10% FBS.

### Real-time PCR and western blotting

IL-6 mRNA levels were determined by qRT-PCR using glyceraldehyde 3-phosphate dehydrogenase (GAPDH) and cyclophilin A (CycA) as reference mRNA. Protein levels were measured by Western blotting as described previously [30]. Immunoblots were analyzed by Odyssey infrared imaging system (LI-COR Biosciences, NE).

### Intracellular Ca^2+^-mobilization assay

MDA-MB-231 cells were serum starved overnight and detached by PBS containing 2 mM EDTA and 0.1% (w/v) fatty acid-free BSA, pH 7.4. Cells were washed and resuspended in Ca^2+^-, Mg^2+^- and phenol red-free Hank’s buffer containing 2.5 mM probenecid and 0.1% (w/v) fatty acid-free BSA. Cells were labeled with 2 mM Fura-2 AM plus 0.02% (w/v) F127, and incubated in the dark at 20 °C for 40 min. Following washing, cells were resuspended in the same buffer at 5x10^5^ cells/ml, and 2 ml of cell suspension was loaded into a quartz cuvette for fluorescence measurement using a fluorometer (C43/2000, PTI, NJ). LPA at 10 μM was used for stimulation. The ratio of emission intensity at 510 nm that was caused by 340 and 380 nm excitation was used to calculate Ca^2+^-mobilization.

### NF-κB translocation assay

MDA-MB-231 and 4T1 cells were cultured in 10-cm dishes and serum starved over night when reach 80% confluent. Doxycycline was added together with the starvation medium. Cells were stimulated on the next day with 5 μM of LPA or 20 ng/ml of TNFα for 0.5, 1 and 2 h. Cells were washed twice with ice-cold PBS followed by adding 0.5 ml of lysis buffer: 10 mM HEPES; pH 7.5, 10 mM KCl, 0.1 mM EDTA, 1 mM dithiothreitol (DTT), 0.5% Nonidet-40 and protease inhibitors. Cells were collected by scraping and kept on ice for 30 min. After centrifuge at 12,000 g for 10 min, the supernatants were collected as a cytoplasmic fraction and the nuclear pellets were washed with lysis buffer for 3 times, and then resuspended in nuclear extraction buffer containing 20 mM HEPES (pH 7.5), 400 mM NaCl, 1 mM EDTA, 1 mM DTT and protease inhibitors and incubated on ice for 30 min. The supernatant was collected by centrifugation at 12,000 g for 15 min at 4 °C as nuclear extract. The level of NF-κB was determined by western blotting.

### Cell proliferation assay in three-dimensional culture

4T1 cells were suspended in DMEM (1.5×10^4^ cells/ml) supplemented with 2% (v/v) growth factor-reduced Matrigel™ and 10% FBSC. Cell suspension (400 μl/well) was put onto the top of a thin layer of Matrigel™ (150 μl/well) in 8-well chamber slides (177402, Thermo Scientific, Burlington, ON, Canada). LPA at 5 μM and doxycycline at 5 μg/ml or 10 μg/ml were applied. Cells were grown for 9 days with daily replacement with fresh medium containing LPA and drugs, and fixed with 4% (w/v) paraformaldehyde. Phase-contrast images were acquired using an AMG EVOS digital inverted microscope (Electron Microscopy Sciences, PA). The average size of cell colonies was measured by ImageJ software.

### Mouse tumor model

A syngeneic orthotopic mouse breast cancer model was established using 4T1 cells as previously reported [30]. All procedures were performed in accordance with the Canadian Council of Animal Care as approved by the University of Alberta Animal Welfare Committee. Female BALB/c mice were given doxycycline 50 mg/kg/day by i.p. injection. Control mice were given saline by i.p. injection. Tumor growth was monitored by two orthogonal caliper measurements and tumor volume was estimated from width^2^ × length/2. After sacrificing the mice, blood and tumors were collected. Lungs were fixed with 10% formalin and then stained with India ink. Nodules on the surface were counted.

### Immunohistochemistry

Tumors were fixed with 10% formalin followed with paraffin embedding and sectioning. Sample treatment and immuno-staining were performed according to the standard procedure. For F4/80 staining, 0.05% trypsin treatment at 37 °C for 10 min was applied for antigen retrieval. For other antibodies, heating with Tris/EDTA, pH 9.0 in a pressure cooker was used for antigen retrieval. Positive staining events for CD45, CD8a, F4/80, Foxp3 and CD31 were selected and counted by ImageJ software. The average counts in 5 fields were calculated for each sample.

### Measurement of plasma LPA concentrations

Mouse blood was collected by cardiac puncture using an EDTA coated syringe. Plasma LPA concentration was measured as described previously [27]. Blood samples were centrifuged at 14,000 rpm for 1 min and plasma was collected. Plasma was treated with isotope labeled C17:0-LPA as internal standards and lipid phosphates were extracted into butan-1-ol. Lysophospholipids were measured by LC/MS with electrospray ionization in the negative ion mode using an Agilent 1200 series LC system coupled to a 3200 QTRAP mass spectrometer (AB Sciex, Concord, ON, Canada). The concentrations of different LPA species (C16:0-LPA, C20:4-LPA, C18:0-LPA and C18:1-LPA) were summed as total LPA concentration in plasma.

### Measurement of ATX activity

ATX activity was measured in 50 μl by the choline released from lysophosphatidylcholine (LPC) as previous reported [27]. Ten μl of plasma was mixed with 15 μl of buffer A (100 mM Tris-HCl, pH 9.0; 500 mM NaCl; 500 mM MgCl_2_; and 0.05% v/v Triton X-100) and preincubated at 37 °C for 30 min. Samples were then mixed with 25 μl of 6 mM C14:0-LPC in buffer A and incubated for 6 h at 37 °C when 20 μl samples were pipetted in duplicate into a 96-well plate and incubated at 37 °C for 20 min with 90 μl/well of buffer C [9.65 ml buffer B (100 mM Tris-HCl, pH 8.5, and 5 mM CaCl_2_), 110 μl of 30 mM TOOS (*N*-ethyl-*N*-(2-hydroxy-3-sulfopropyl)-3-methylaniline, sodium salt, dehydrate; Dojindo Molecular Technologies, Rockville, MD, USA), 110 μl of 50 mM 4-aminotipyrine, 6.6 μl of 1000 U/ml horseradish peroxidase, and 110 μl of 300 U/ml choline oxidase]. Choline formation was measured at 550 nm. Activity measurements were normalized to protein content.

### Multiplex cytokine/chemokine measurements

Cytokines, chemokines, and growth factors were analyzed by Eve Technologies Corp. (Calgary, AB, Canada), with a Milliplex Mouse Cytokine/Chemokine 32-plex kit (Millipore, MO), according to the manufacturer’s protocol, on a Luminex 100 system (Luminex, Austin, TX). Tissue specimens (10–25 mg) were homogenized in 200 μl of 20 mM Tris-HCl (pH 7.5) buffer with 0.5% Tween 20, 150 mM NaCl, and protease inhibitors and centrifuged for 10 min at 4 °C, and the supernatant was transferred to a fresh tube. Protein content was measured using the bicinchoninic acid protein assay (Thermo Fisher Scientific; IL). For supernatant analysis, conditioned media were centrifuged before multiplex analysis. Measurements were normalized to the cell protein. Plasma samples were diluted with an equal volume of PBS before analysis.

### Separation of mouse PBMCs and the transwell migration assay

Blood was collected by cardiac puncture in EDTA-coated tubes from 10 mice. Blood was diluted at 1:1 (v/v) with tricine-buffered saline (TBS): 0.85% NaCl, 10 mM Tricine-NaOH, pH7.0. A density barrier was prepared by mixing OptiPrep™ with TBS and water at volume ratio of 45:155:31 respectively. One ml of the density barrier was added in a 15-ml centrifuge tube followed by adding 2 ml of diluted blood on the top of it. Tubes were centrifuged at 700 g for 20 min at 20 °C. The PBMC band was collected at the interface. Contaminating red blood cells were removed using a red blood cell lysis buffer consisting of: 155 mM NH_4_Cl, 10 mM KHCO_3_, 0.1 mM EDTA, pH 7.3. The PBMC pellet was washed twice with RPMI medium. PBMCs were cultured in RPMI with 0.1% BSA. Cells were pre-treated with 5 μg/ml doxycycline for 12 h before the experiment. PBMCs were labeled with 5 μM calcein-AM at 37 °C for 30 min. Cells were washed 3 times with RPMI and resuspended in RPMI with 0.1% BSA at 5×10^6^ cells/ml. The transwell assay was performed using a 96-well Boyden chamber as reported previously [30]. LPA at 1 μM or CCL2 at 100 ng/ml were added in the lower chambers and 100 μl of cell suspension was added into the upper chamber. The chambers were incubated at 37 °C in a tissue culture incubator for 90 min. Cells that migrated into the lower chamber were quantified with a plate reader (Fluoroskan Ascent, ThermoFisher Scientific, Burlington, ON, Canada) at Ex488/Em510.

### Statistical analysis

Results were analyzed by Student’s *t*-test or by ANOVA for multiple comparisons followed by Student-Newman-Keuls (SNK) test. *p* < 0.05 was considered statistically significant.

## Additional files

Additional file 1: Effects of doxycycline (Dox) on tumor growth, metastasis, and leukocyteinfiltration. (PPTX 2548 kb)
Additional file 2: Cytokine levels in plasma of the BALB/c mice with tumor. (PPTX 274 kb)
Additional file 3: Effects of doxycycline (Dox) on NF-κB activity. (PPTX 102 kb)
Additional file 4: Effects of Doxycycline (Dox) on Ki-67 expression. (PPTX 706 kb)

## Abbreviations

ATX: Autotaxin
CCL: Chemokine (C-C motif) ligand
CXCL: Chemokine (C-X-C motif) ligand
G-CSF: Granulocyte colony-stimulating factor
IL: Interleukin
LIF: Leukemia inhibitory factor
LPA: Lysophosphatidic acid, which at physiological pH is in a salt form, lysophosphatidate
LPP: Lipid phosphate phosphatase
LPS: Lipopolysaccharide
PBMC: Peripheral blood mononuclear cells
TAM: Tumor-associated macrophages
VEGF: Vascular endothelial growth factor
