# Supplementary material for: Doxycycline attenuates breast cancer related inflammation by decreasing plasma lysophosphatidate concentrations and inhibiting NF-κB activation
Source: Mol Cancer. 2017 Feb 8;16:36. doi: 10.1186/s12943-017-0607-x (PMC5299726; doi:10.1186/s12943-017-0607-x)
Supplement: Additional file 1: — Effects of doxycycline (Dox) on tumor growth, metastasis, and leukocyteinfiltration. (PPTX 2548 kb) [file 12943_2017_607_MOESM1_ESM.pptx]

## Slide 1
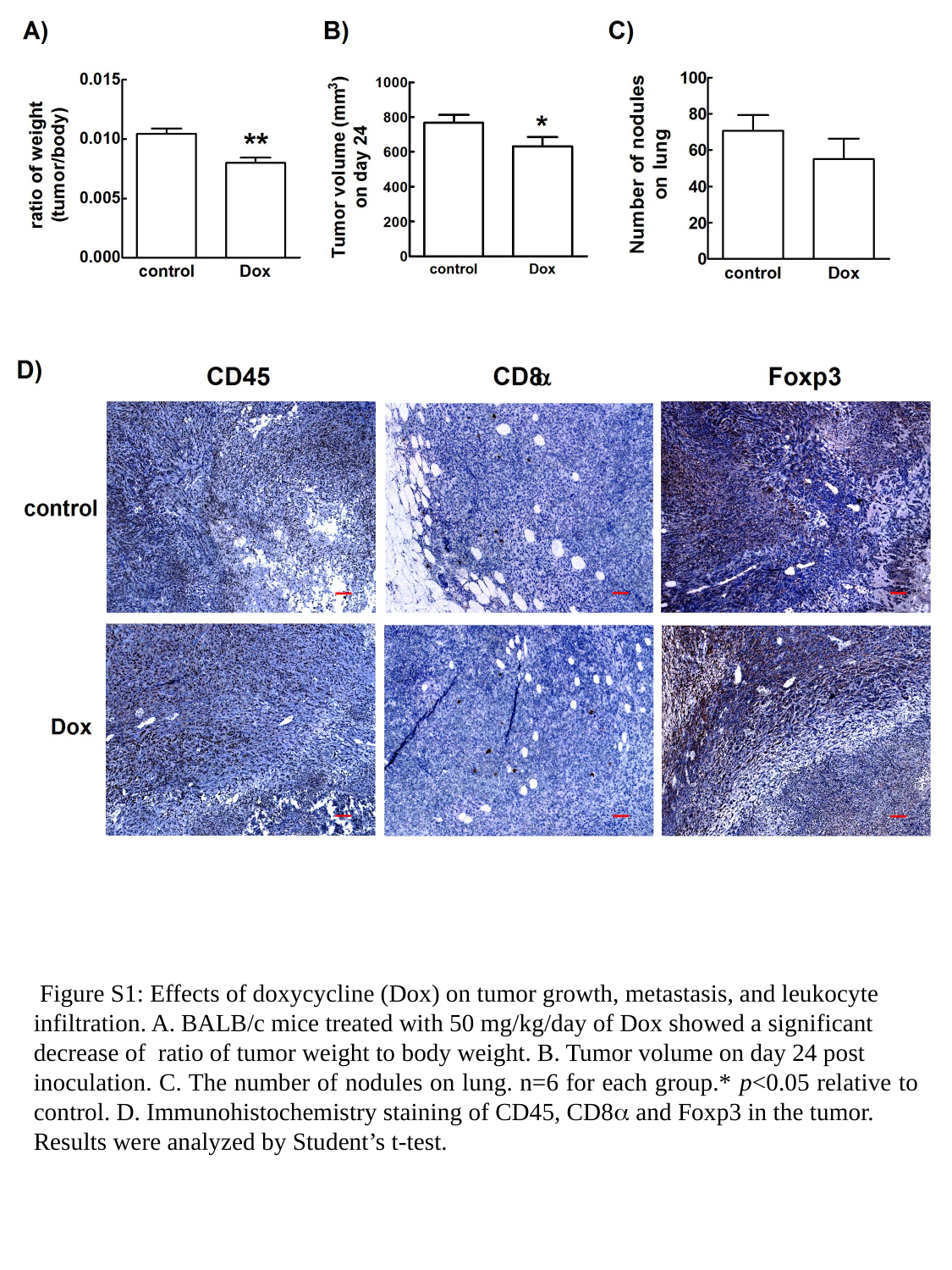

Figure S1: Effects of doxycycline (Dox) on tumor growth, metastasis, and leukocyte
infiltration. A. BALB/c mice treated with 50 mg/kg/day of Dox showed a significant
decrease of ratio of tumor weight to body weight. B. Tumor volume on day 24 post
inoculation. C. The number of nodules on lung. n=6 for each group.* p<0.05 relative to control. D. Immunohistochemistry staining of CD45, CD8a and Foxp3 in the tumor.
Results were analyzed by Student’s t-test.
