# Supplementary material for: Doxycycline attenuates breast cancer related inflammation by decreasing plasma lysophosphatidate concentrations and inhibiting NF-κB activation
Source: Mol Cancer. 2017 Feb 8;16:36. doi: 10.1186/s12943-017-0607-x (PMC5299726; doi:10.1186/s12943-017-0607-x)
Supplement: Additional file 2: — Cytokine levels in plasma of the BALB/c mice with tumor. (PPTX 274 kb) [file 12943_2017_607_MOESM2_ESM.pptx]

## Slide 1
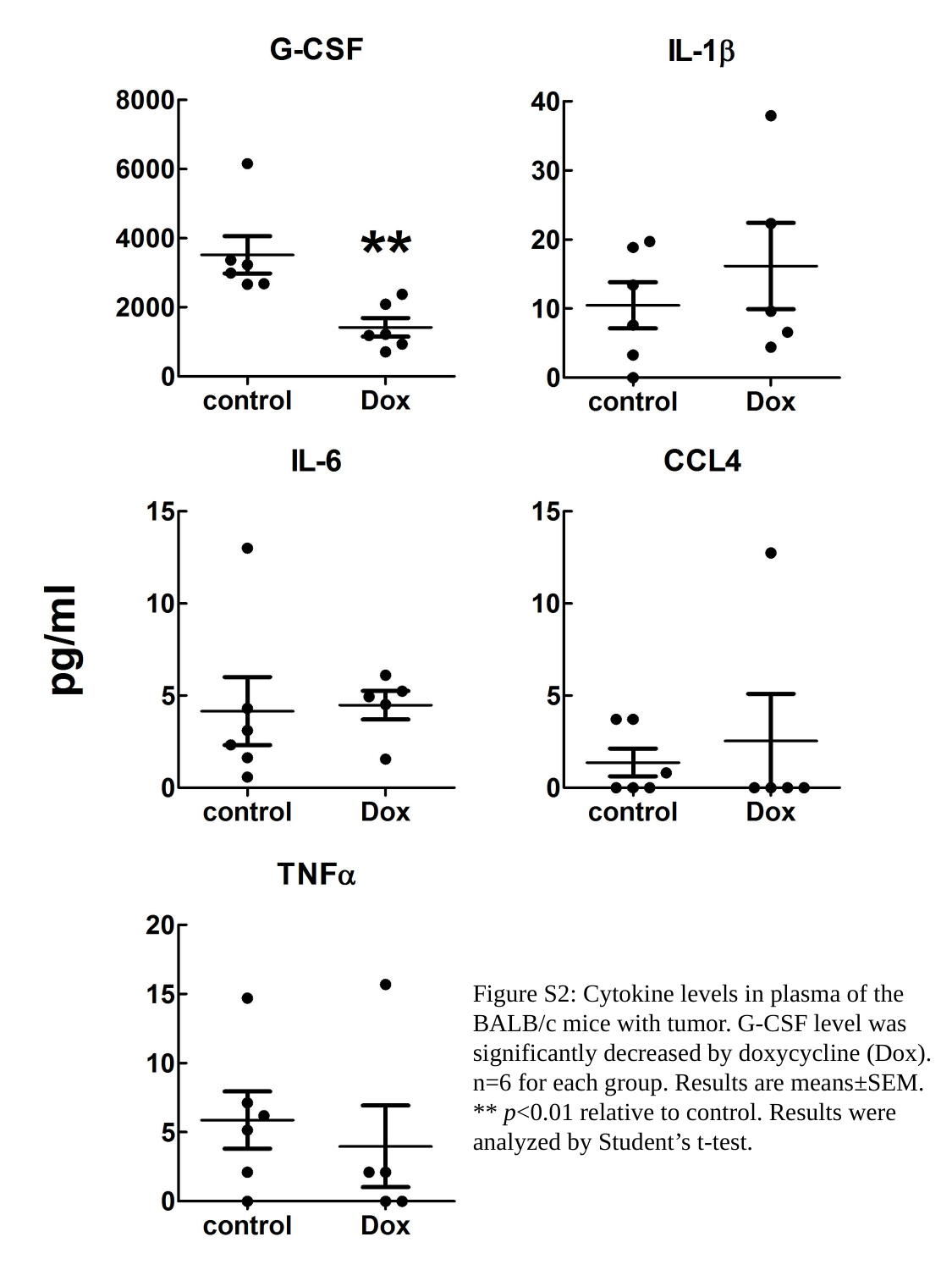

Figure S2: Cytokine levels in plasma of the
BALB/c mice with tumor. G-CSF level was
significantly decreased by doxycycline (Dox).
n=6 for each group. Results are means±SEM.
** p<0.01 relative to control. Results were
analyzed by Student’s t-test.
