# Supplementary material for: Doxycycline attenuates breast cancer related inflammation by decreasing plasma lysophosphatidate concentrations and inhibiting NF-κB activation
Source: Mol Cancer. 2017 Feb 8;16:36. doi: 10.1186/s12943-017-0607-x (PMC5299726; doi:10.1186/s12943-017-0607-x)
Supplement: Additional file 3: — Effects of doxycycline (Dox) on NF-κB activity. (PPTX 102 kb) [file 12943_2017_607_MOESM3_ESM.pptx]

## Slide 1
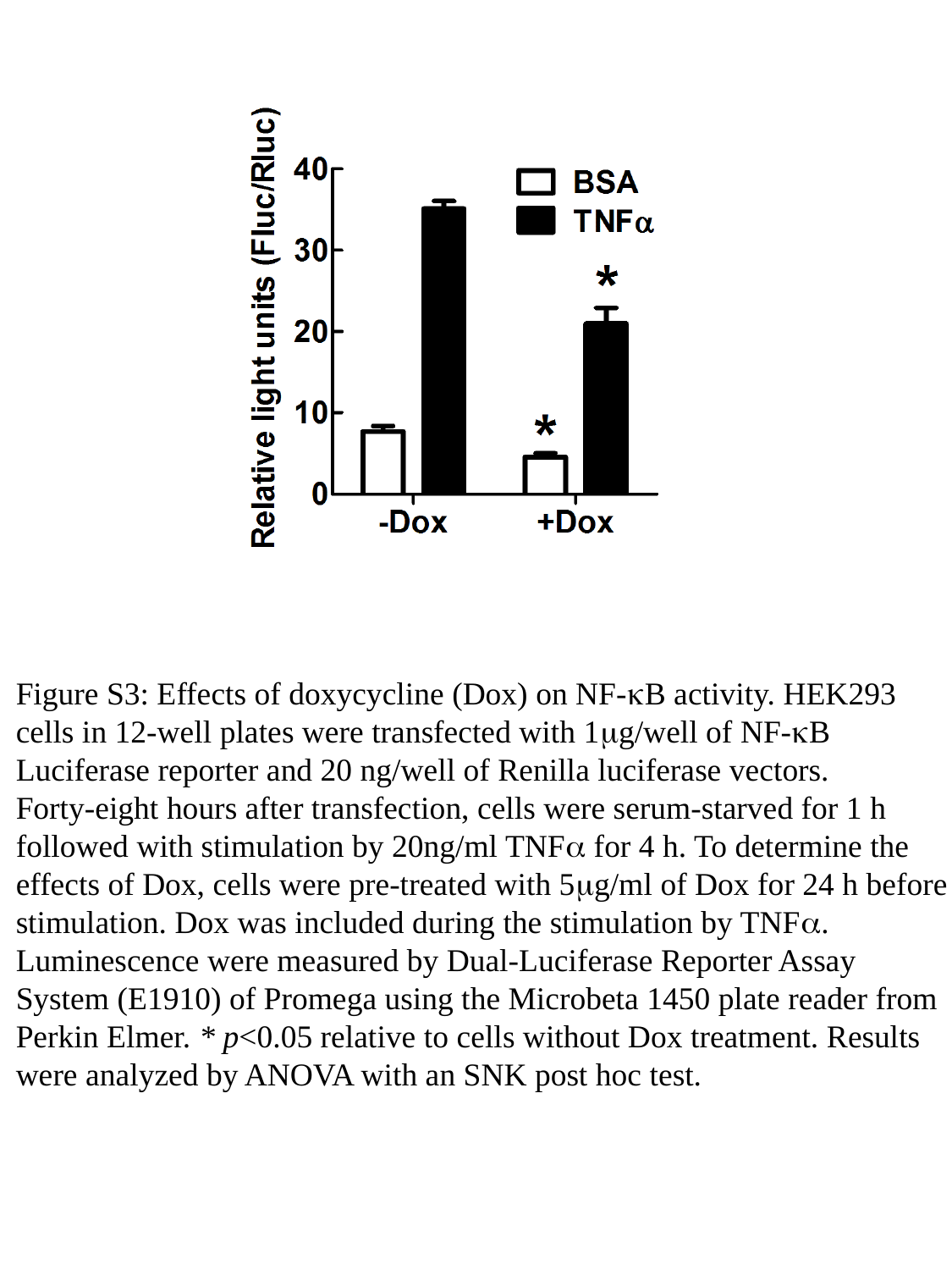

Figure S3: Effects of doxycycline (Dox) on NF-kB activity. HEK293
cells in 12-well plates were transfected with 1mg/well of NF-kB
Luciferase reporter and 20 ng/well of Renilla luciferase vectors.
Forty-eight hours after transfection, cells were serum-starved for 1 h
followed with stimulation by 20ng/ml TNFa for 4 h. To determine the
effects of Dox, cells were pre-treated with 5mg/ml of Dox for 24 h before
stimulation. Dox was included during the stimulation by TNFa.
Luminescence were measured by Dual-Luciferase Reporter Assay
System (E1910) of Promega using the Microbeta 1450 plate reader from
Perkin Elmer. * p<0.05 relative to cells without Dox treatment. Results
were analyzed by ANOVA with an SNK post hoc test.
