# Supplementary material for: Doxycycline attenuates breast cancer related inflammation by decreasing plasma lysophosphatidate concentrations and inhibiting NF-κB activation
Source: Mol Cancer. 2017 Feb 8;16:36. doi: 10.1186/s12943-017-0607-x (PMC5299726; doi:10.1186/s12943-017-0607-x)
Supplement: Additional file 4: — Effects of Doxycycline (Dox) on Ki-67 expression. (PPTX 706 kb) [file 12943_2017_607_MOESM4_ESM.pptx]

## Slide 1
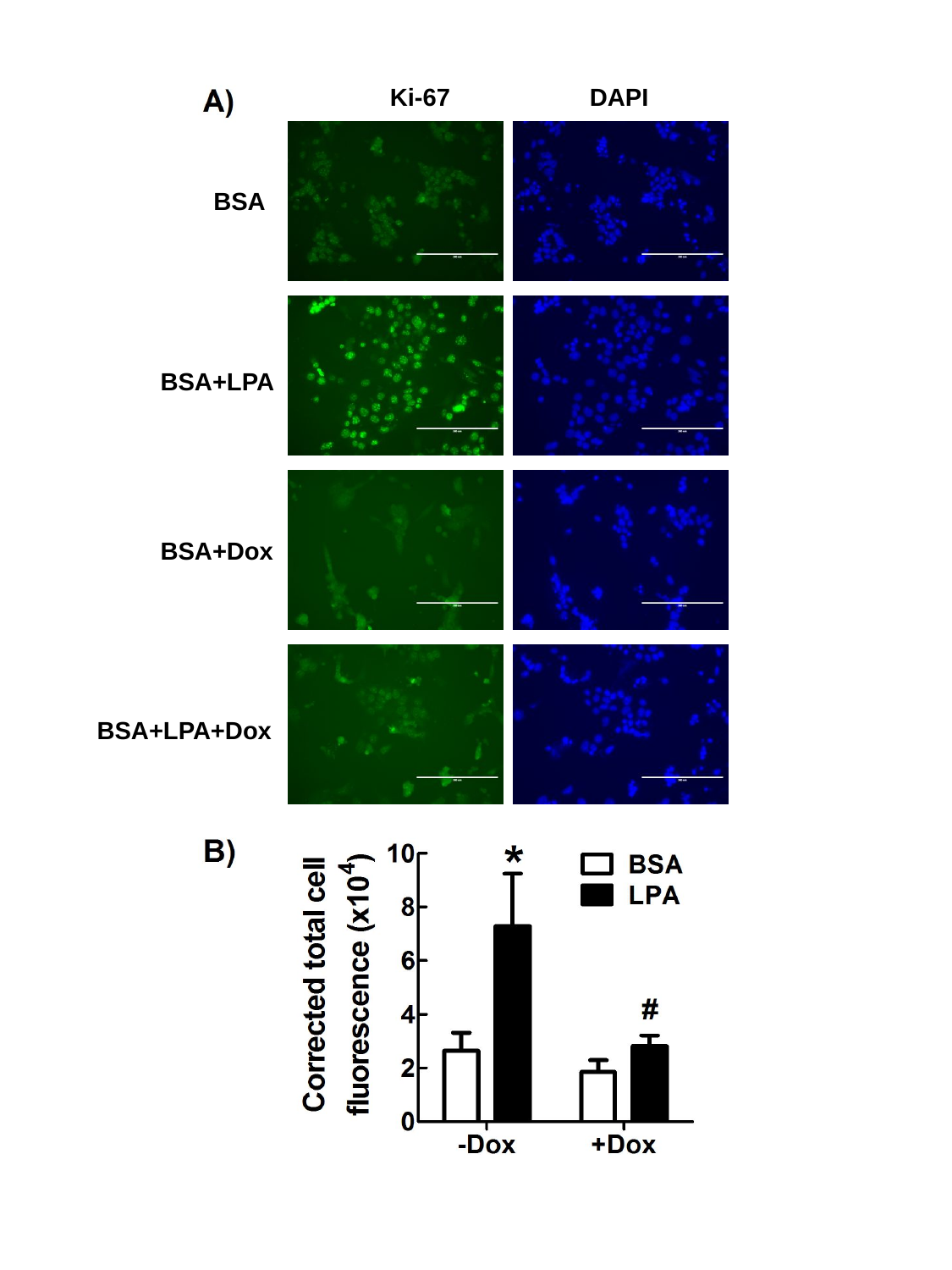

Ki-67 DAPI
BSA
BSA+LPA
BSA+Dox
BSA+LPA+Dox

## Slide 2
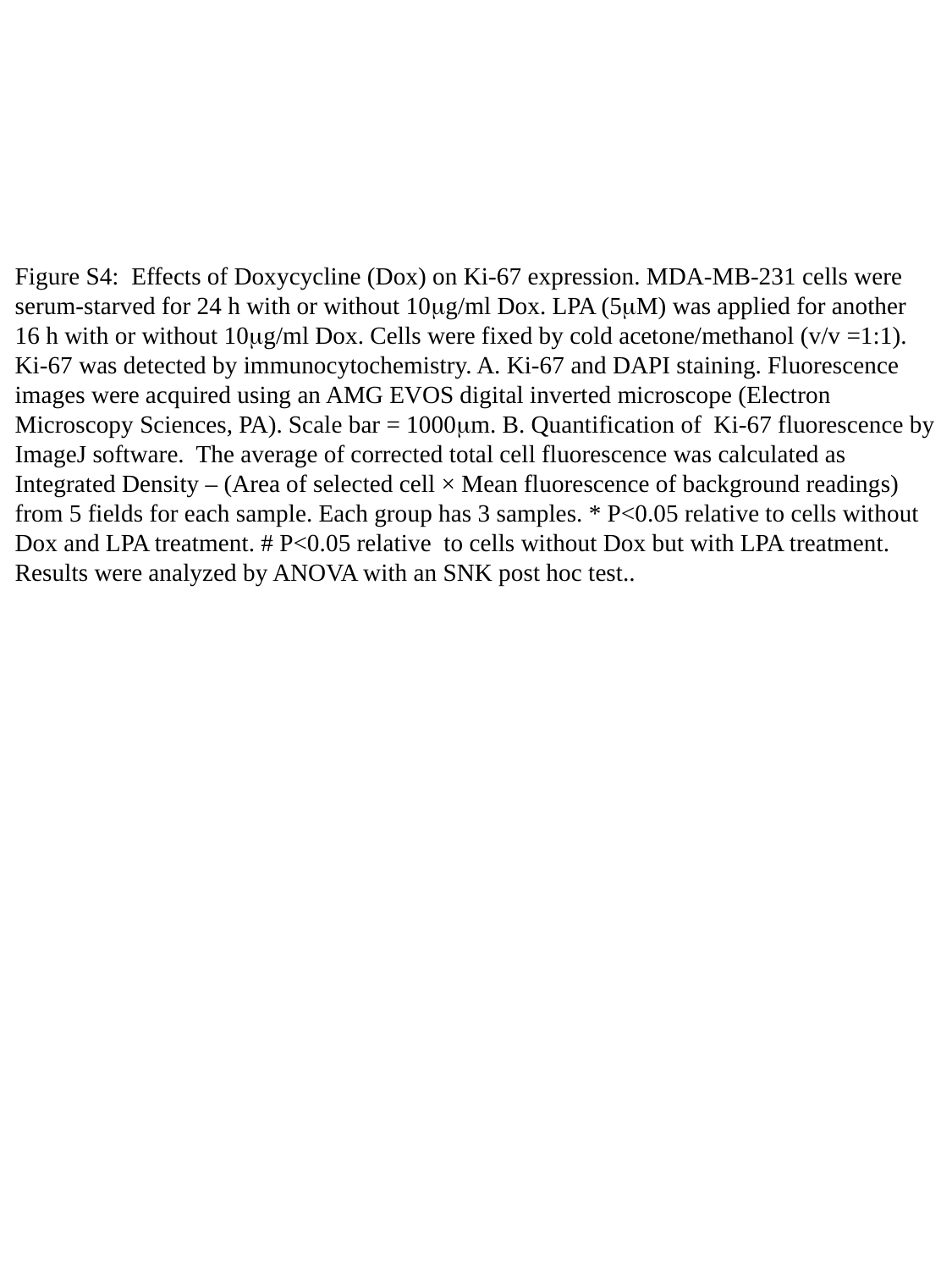

Figure S4: Effects of Doxycycline (Dox) on Ki-67 expression. MDA-MB-231 cells were
serum-starved for 24 h with or without 10mg/ml Dox. LPA (5mM) was applied for another
16 h with or without 10mg/ml Dox. Cells were fixed by cold acetone/methanol (v/v =1:1).
Ki-67 was detected by immunocytochemistry. A. Ki-67 and DAPI staining. Fluorescence
images were acquired using an AMG EVOS digital inverted microscope (Electron
Microscopy Sciences, PA). Scale bar = 1000mm. B. Quantification of Ki-67 fluorescence by
ImageJ software.  The average of corrected total cell fluorescence was calculated as
Integrated Density – (Area of selected cell × Mean fluorescence of background readings)
from 5 fields for each sample. Each group has 3 samples. * P<0.05 relative to cells without
Dox and LPA treatment. # P<0.05 relative to cells without Dox but with LPA treatment.
Results were analyzed by ANOVA with an SNK post hoc test..
